# 聚酰胺-胺树状大分子功能化离子型分离介质研究进展

**DOI:** 10.3724/SP.J.1123.2024.06002

**Published:** 2025-07-08

**Authors:** Ding ZHOU, Dandan GUO, Yan ZHU

**Affiliations:** 1.宁波卫生职业技术学院，浙江 宁波 315000; 1. Ningbo College of Health Sciences，Ningbo 315000，China; 2.宁波大学新药技术研究院，浙江 宁波 315211; 2. Institute of Drug Discovery Technology，Ningbo University，Ningbo 315211，China; 3.浙江大学化学系，浙江 杭州 310028; 3. Department of Chemistry，Zhejiang University，Hangzhou 310028，China

**Keywords:** 聚酰胺-胺, 树状大分子, 离子相互作用, 色谱固定相, 吸附剂, 综述, polyamide-amine （PAMAM）, dendrimer, ionic interaction, chromatography stationary phase, adsorbent, review

## Abstract

树状大分子作为一种拥有独特树形结构的新型功能高分子材料，正逐渐被应用于色谱前处理材料的制备以及色谱固定相填料的修饰中。其中，聚酰胺-胺（PAMAM）树状大分子因其广泛的使用和显著的特性，成为该领域内备受关注的材料之一。在制备与修饰PAMAM功能化分离材料的过程中，PAMAM树状大分子所含的大量功能化末端基团为分离材料表面带来了丰富的相互作用位点；PAMAM树状大分子的超支化结构使得在有限的基质表面上能实现更高的接枝效率；同时，PAMAM树状大分子可控的树状结构有效调控了分离材料表面的结构。特别是整数代的PAMAM树状大分子，其末端氨基官能团经过质子化或季铵化修饰后，会带上高密度正电荷，从而与带负电荷的阴离子物质产生良好的静电相互作用，展现出对带电物质良好的富集与分离性能。本文基于整数代PAMAM树状大分子的结构特点，从离子相互作用机理出发，总结了经过质子化或季铵化修饰的PAMAM树状大分子在离子型吸附剂及离子色谱固定相填料制备领域的研究进展。同时，对这类材料未来的发展潜力及应用前景进行了展望。

## Abstract (en)

Recent years have witnessed the gradual use of dendrimers to prepare and modify various separation materials， including adsorbents and chromatographic stationary phases. Polyamide-amine （PAMAM） has become one of the most widely used dendritic materials because it is simple to prepare， inexpensive， hypotoxic， and has excellent performance characteristics. PAMAM is advantageous as a modification material for adsorbents and chromatographic stationary phases compared to other traditional materials. Firstly， the large number of terminal groups on a PAMAM dendrimer provide abundant interaction sites on the surface of the functional matrix， which is beneficial for delivering superior adsorption capacity and separation selectivity. In addition， the hyperbranched structure of a PAMAM dendrimer facilitates higher grafting efficiency on the limited surface area of the matrix while simultaneously improving the uniformity of the functional groups grafted on the surface. Moreover， the controllable structure of PAMAM dendrimer effectively regulates the surface structure of separated materials. Moreover， the terminal amino functional groups of integer-generational PAMAM dendrimer are capable of carrying a high density of positive charges following protonation or quaternization， thereby facilitating good electrostatic interactions with negatively charged anionic substances that lead to excellent enrichment and separation effects. Based on the structural characteristics of integer generation PAMAM dendrimer and the mechanism of ionic interaction， the research progress of protonated or quaternized PAMAM dendrimer in the preparation of ionic adsorbents and ion chromatography stationary phases was summarized in this paper. Their future development potential and applications prospects are also discussed.

离子相互作用是带电粒子间的相互吸引或相互排斥作用，在化合物的分离与分析过程中发挥着至关重要的作用。例如，在基于静电相互作用的固相萃取分离中，通过利用不同化合物与吸附剂间的静电吸附作用差异，实现目标化合物的有效富集与分离^［1，2］^。在基于离子交换原理的离子色谱法中，通过分析物与离子交换树脂间离子交换强度的不同，来实现离子的有效分离^［3］^。在上述过程中，那些能够提供离子相互作用的分离材料（吸附剂和色谱固定相）是不可或缺的关键因素。作为分析化学领域中重要的分离材料，离子型吸附剂和离子色谱固定相填料具有类似的结构，二者均由固定相基质和表面官能团组成。离子色谱固定相的早期形式主要以硅胶基质为主，这种基质具有出色的刚性以及易于调控的物理特性，如粒径、孔结构和比表面积等^［4］^，然而，其pH耐受性相对有限^［5，6］^，因此后来逐渐被聚合物基质所取代。聚合物基质通常由苯乙烯、二乙烯基苯和丙烯酸甲酯等有机单体^［7-9］^通过交联聚合的方式制备而成。与传统的硅胶基质相比，聚合物基质展现出更宽的pH耐受范围，因此被广泛用作离子色谱固定相基质。与离子色谱固定相填料相比，离子型吸附剂的基质选择范围更加广泛。除了硅胶和聚合物微球外，还可选择金属氧化物、碳量子点、金属有机框架以及共价有机框架等各种新型材料^［10-14］^。除了基质的选择之外，基质表面的功能化基团对离子型吸附剂和离子色谱固定相的性能同样具有至关重要的影响。具体而言，针对阳离子物质的吸附与分离，所使用的吸附剂和色谱填料表面主要修饰有带负电荷的官能团，如羧酸和磺酸等^［15，16］^；而对于阴离子物质的吸附与分离，材料表面的带电官能团则主要为质子化的氨基官能团^［17］^以及季铵化的氨基官能团^［18］^。

聚酰胺-胺（PAMAM）树状大分子是一种以乙二胺为核心、酰胺键为重复结构单元的一类树状大分子材料。整数代PAMAM树状大分子拥有大量易于质子化的末端氨基官能团，基于该特性，本课题组^［17，19-25］^近年来在PAMAM离子型吸附剂和离子色谱固定相填料的制备和应用研究方面取得了阶段性的进展。本综述将围绕PAMAM树状大分子功能化离子型吸附剂和离子色谱固定相填料的制备技术及其应用展开，重点探讨整数代PAMAM树状大分子在基于离子相互作用的分离材料制备中的关键作用；深入分析PAMAM树状大分子所展现的独特优势及潜在局限性；同时，本综述还将展望PAMAM树状大分子在该领域的未来发展趋势，旨在为相关领域的研究与应用提供有益的参考与启示。

## 1 PAMAM树状大分子概述

树状大分子是20世纪30年代开发出来的一类新型功能高分子材料，具有完美的树形结构^［26，27］^。这类材料能够在分子水平上精确控制其大小、形状、结构以及功能基团的设计。它们的高度支化结构、均匀的单分散性、内部宽敞的空腔以及丰富的末端活性基团赋予了树状大分子独特的性质和功能，如良好的分散性和生物相容性、低毒性、优异的电学和光学性能，以及易于修饰等特点^［28］^。树状大分子的制备主要通过发散法、收敛法以及发散-收敛法这3种方法实现^［26，29-31］^。由于在制备过程中能够精确调控分子链，树状大分子展现出完美的三维空间结构。

在众多树状大分子材料中，PAMAM因其合成工艺简便且成本低廉，已成为迄今为止应用最为广泛的树状大分子材料之一。PAMAM树状大分子以乙二胺为核心，通过发散法，利用乙二胺和丙烯酸甲酯进行交替的Michael加成和酰胺化反应制备而成。在制备过程中，每一次交替反应都会生成一组重复的结构单元，每一个重复的结构单元被称为一代（G）。每半代树状大分子的末端以酯基官能团结束，而每整数代的树状大分子末端都含有大量的氨基官能团（见图1）。随着PAMAM树状大分子代数的增加，其末端官能团的数量呈指数级增长（表1）。经过质子化或季铵化修饰后，以氨基为末端官能团的整数代PAMAM树状大分子末端会带有大量正电荷，从而能够为带负电荷的阴离子物质提供充足的离子相互作用结合位点（图2）。基于这种相互作用机制，PAMAM功能化的离子型色谱分离材料在带负电粒子的固相萃取和色谱分离领域展现出了广阔的应用前景。

**图1:**
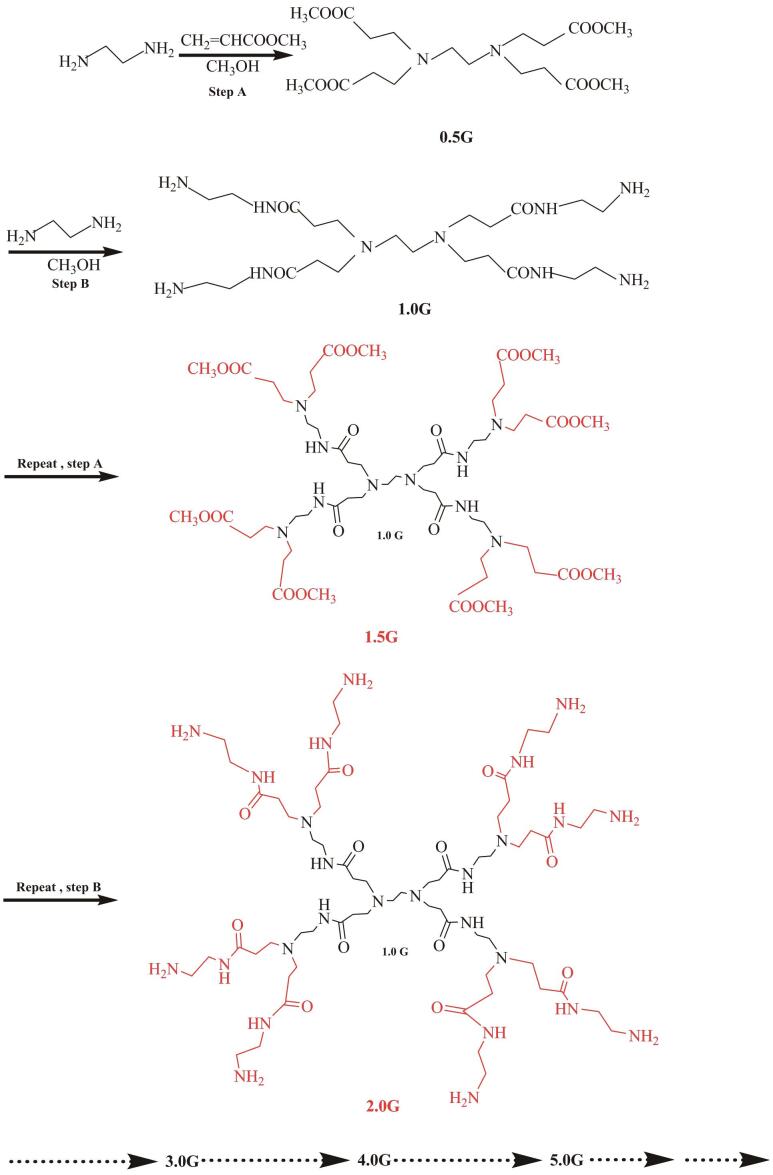
PAMAM树状大分子的合成路线

**表1:** 各个代数PAMAM树状大分子的分子式、相对分子质量和末端官能团数量

Algebra	Molecular formula	Relative molecular mass	Number of terminal functional groups
0G	C_2_H_8_N_2_	60	2
1.0G	C_22_H_48_O_4_N_10_	516	4
2.0G	C_62_H_128_O_12_N_26_	1428	8
3.0G	C_142_H_288_O_28_N_58_	3252	16
4.0G	C_302_H_608_O_60_N_122_	6900	32
5.0G	C_622_H_1248_O_124_N_250_	14196	64
6.0G	C_1262_H_2528_O_252_N_506_	28788	128

**图2:**
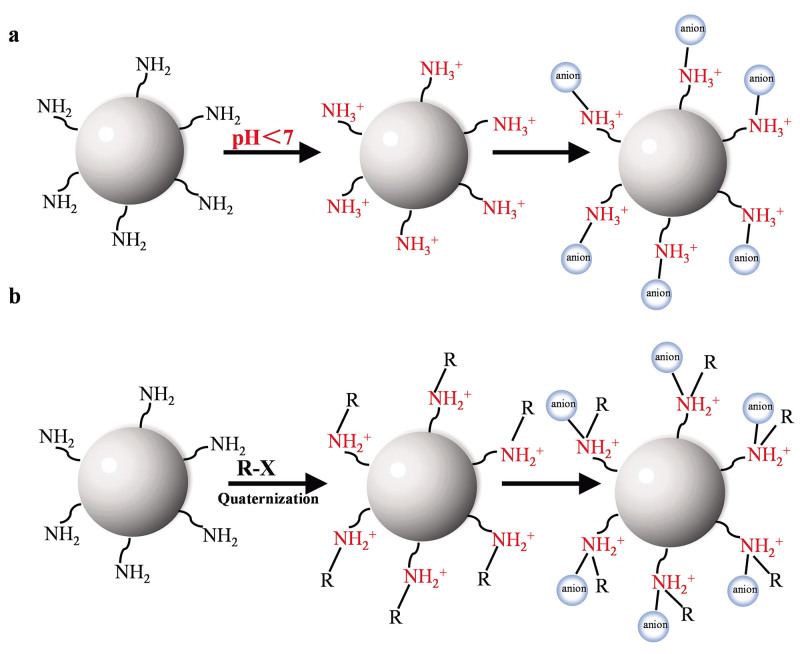
整数代PAMAM树状大分子对阴离子物质的吸附机理

## 2 PAMAM功能化离子型吸附剂的应用

目前的文献研究表明^［17，32-34］^，基于离子相互作用的PAMAM吸附材料在无机阴离子、酸根离子、有机染料、有机农药和生物活性物质的吸附中展现出优异性能，表现出广阔的应用前景。

### 2.1 无机阴离子及酸根离子的吸附应用

在众多阴离子污染物中，一些无机阴离子及酸根离子对人类的身体健康会造成不可逆转的危害。例如，硝酸盐可诱发婴儿高铁血红蛋白血症（即蓝婴综合征），促进致癌物亚硝胺的生成并引发高血压^［35］^；人体接触砷酸盐会导致周围神经病变及皮肤癌、膀胱癌、肺癌等多器官癌变，同时引发周围血管疾病；人体长期接触铬酸盐则会导致肺癌。因此，吸附和去除这些无机阴离子及酸根离子，对于环境保护及人类生命健康保障十分重要。针对这一需求，多种PAMAM功能化的阴离子吸附材料得到了广泛的开发和应用。Hassan等^［36］^将磁性纳米粒子固定于PAMAM/蒙脱土（M@PAMAM/MMT）材料中，以清除硝酸盐。根据离子色谱分析结果，所制备的纳米复合物对NO_3_^-^的清除效率可达83%，其清除机理主要依赖于NO_3_^-^与PAMAM末端基团（NH_3_^+^）之间的静电相互作用。Harinath等^［37］^通过对CoFe_2_O_4_纳米颗粒表面进行硅烷化修饰，进而在其表面接枝PAMAM树状聚合物，成功合成了尖晶石铁氧体（DFSF）材料。DFSF凭借其表面丰富的吸附位点，能够在5 min内实现对Pt（Ⅳ）和Pd（Ⅱ）的快速吸附；在强酸性条件下，DFSF对Pt（Ⅳ）和Pd（Ⅱ）的吸附量分别为404 mg/g和523 mg/g。DFSF的吸附机制主要归因于酸性条件下PAMAM表面氨基的质子化效应，使得DFSF与溶液中的［PtCl_6_］^2-^和［PdCl_4_］^2-^产生静电相互作用。此外，该材料具有优异的循环稳定性及低饱和磁化强度，并且易于从水溶液中分离，因此有望成为一种用于贵金属吸附回收的新型材料。有研究人员用3.0代（3.0G）PAMAM聚合物吸附水中的Cr（Ⅵ）^［38］^，当溶液pH值从7.0降至2.0时，Cr（Ⅵ）的去除率从34.98%上升至97.02%。这一现象的主要原因是PAMAM在酸性条件下更容易发生质子化，而Cr（Ⅵ）在水溶液中主要以HCrO_4_^-^、Cr_2_O_7_^2-^和CrO_4_^2-^等多种形态存在，酸性条件下PAMAM与Cr（Ⅵ）的阴离子形态之间能够产生离子相互作用，从而提高了吸附效率。

### 2.2 有机染料及有机农药的吸附应用

污水中的有机染料或有机农药也同样会破坏水生生物的生长发育，并通过食物链逐级放大，最终导致人类肾脏、生殖系统、肝脏、大脑和中枢神经系统的功能障碍^［35，39］^。PAMAM功能化吸附材料对水溶液中的有机阴离子污染物表现出良好的富集效果。Wang等^［40］^制备了壳聚糖（MCS）/PAMAM微粒子作为磁性吸附剂，用于去除自制标准液中的活性蓝21（RB 21）染料。研究发现，当溶液pH低于8.5时，MCS/PAMAM表面发生质子化而带正电，从而有利于RB 21的吸附。经过3次循环使用后，该材料仍能保持41.64%的吸附回收率。Fard等^［41］^利用三步法制备了PAMAM接枝的*α*-Fe_2_O_3_纳米纤维，并将其作为新型吸附剂，用于刚果红80和酸性红18的吸附。研究发现，在pH=3的条件下，该改性纤维表现出很高的吸附能力，对刚果红80和酸性红18的吸附量分别可达1 428.57 mg/g和1 250 mg/g。基于相似的机理，近期Afshar等^［42］^和Zhang等^［43］^分别合成了PAMAM-CuFe_2_O_4_磁铁矿纳米颗粒和MCS原位生长PAMAM气凝胶（CTS-G*x* PAMAM（*x*=0，1，2，3））两种吸附材料。研究结果表明，这两种材料对芹菜素红（ARS）、亮绿（BG）、玫瑰红（RB）和日落黄（SY）染料都具有良好的吸附效果。此外，本课题组^［17］^通过将不同代数的PAMAM树状大分子接枝到聚合物微球表面，成功制备了一种用于吸附水样中草甘膦分子的新型材料。研究表明，PAMAM良好的亲水性显著提高了聚合物微球在溶液中的分散性，从而提高了吸附效率。在pH=3的条件下，吸附剂表面的质子化氨基可通过静电作用吸附草甘膦分子（图3），并在5 min内快速达到吸附平衡。经NaOH洗脱后，该吸附材料可循环使用5次以上。值得注意的是，通过调控PAMAM树状大分子的代数，可以实现对草甘膦吸附量的高度调控，且该材料对实际样品中的草甘膦也表现出良好的吸附效果。

**图3:**
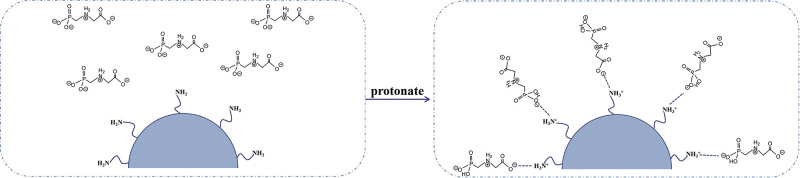
草甘膦分子的静电吸附过程^［17］^

### 2.3 生物活性物质的吸附应用

磷元素被认为是探索原始生命过程中的重要信号。许多生物过程是通过磷酸化和去磷酸化来调控的，其中磷酸化生物分子在这些过程中起着重要的作用。本课题组^［19］^充分利用PAMAM树状大分子易于质子化的化学特性，合成了基于聚合物基质的PAMAM复合材料，并将其用于磷酸肽的高效富集。基于PAMAM树状大分子与磷酸基团之间的相互作用，溶液中的*N*-磷酸基肽可在30 s内实现高效吸附和回收。通过与高效液相色谱联用，我们成功实现了饮用水中磷酰化丙谷二肽含量的精确测定。该研究证实了PAMAM功能化离子型吸附剂在磷酸化生物分子富集与分析领域具有广阔的应用前景。

## 3 PAMAM在离子色谱固定相填料制备中的应用

得益于聚合物基质优异的稳定性，目前报道的离子色谱固定相填料大多采用聚合物微球作为基质载体，并通过在聚合物表面采用表面离子化^［44-46］^、化学接枝^［47-50］^、乳胶附聚^［51-53］^、超支化修饰^［54-56］^以及上述多种修饰方法相结合的方式^［57-59］^来对基质进行进一步修饰（图4）。近年来，随着材料科学领域的快速发展，各类新型功能材料在离子色谱固定相表面修饰中的应用日益广泛。其中，受超支化修饰方法的启发，具有独特树形结构的树状大分子成为一类重要的修饰材料。基于上述离子色谱固定相填料的制备方法，本课题组采用化学接枝和乳胶附聚技术，将PAMAM树状大分子材料应用于新型离子色谱固定相填料的制备中，并取得了一些阶段性的成果^［22，24，25，60］^。

**图4:**
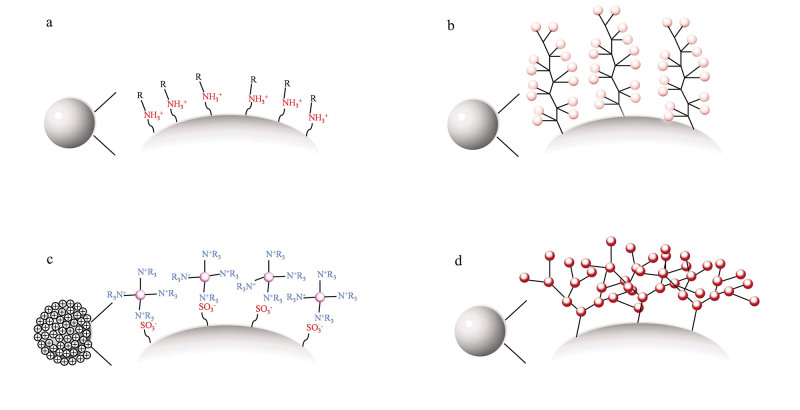
离子色谱固定相填料的常见制备方法

将PAMAM树状大分子应用于离子色谱固定相修饰不仅能够继承超支化修饰方法的优势，还可有效解决传统超支化修饰过程中因空间位阻导致的结构缺陷问题^［60］^。然而，目前PAMAM树状大分子在离子色谱固定相开发领域的研究仍处于初步探索阶段，相关研究成果的报道较为有限。2016年，本课题组^［22］^首次将不同代数的PAMAM树状大分子材料引入离子色谱固定相表面。通过1，4-丁二醇二缩水甘油醚（BDDE）对PAMAM末端氨基进行季铵化修饰，成功制备了以聚苯乙烯-二乙烯基苯-甲基丙烯酸缩水甘油酯（PS-GMA）微球为基质的PAMAM树状大分子接枝型离子色谱填料。该PAMAM修饰的色谱填料既保持了聚合物基质良好的热稳定性和化学稳定性，同时还能提供离子色谱所需的大量季铵基团。色谱流出曲线显示，在同一种聚合物基质表面分别接枝1.0G PAMAM和2.0G PAMAM所得到的离子交换色谱柱，其柱容量分别为0.064 8和0.084 3 mmol/柱。这一发现证实，通过精确调控PAMAM树状大分子的代数，可以实现对离子交换色谱柱容量的有效调控。然而，尽管该色谱固定相填料对常见阴离子和糖类等物质表现出良好的分离性能，但其理论塔板数相对较低，其柱效仍有待进一步提高。随后，我们将氧化石墨烯（GO）引入到PS-GMA微球中，并通过PAMAM树状大分子和BDDE试剂进行功能化修饰，成功制备出高容量的PAMAM接枝型氧化石墨烯杂化阴离子交换剂。与直接在PS-GMA微球上接枝相比，GO杂化的PAMAM接枝型阴离子色谱固定相的柱容量提升了近120%。研究还发现，GO的引入不仅改善了基质表面的亲水性和稳定性，提高了柱效，同时还增强了固定相基质的热稳定性，这为未来开发高温、高压色谱柱奠定了重要基础^［24］^。本课题组^［25］^通过水热法成功制备了纳米碳质球（HNCS），并采用PAMAM树状大分子对其进行接枝和季铵化修饰，开发出阴离子交换色谱（AEC）填料（图5a）。研究表明，在不增加柱长（15 cm）的条件下，与表面化学接枝法相比，树状大分子材料能够在HNCS表面接枝更多的离子交换位点，从而有效提升填料的柱容量。在等度洗脱条件下，7种常见的无机阴离子和6种有机酸在9 min内得到了快速分离（图5b）。该ACE填料的理论塔板数和不对称系数分别为54 000~79 800块/m和1.02~1.12。与已报道的同类填料及商业化产品相比，该填料展现出更优异的色谱性能，包括更高的柱效、更好的峰对称性以及更短的分析时间。

**图5:**
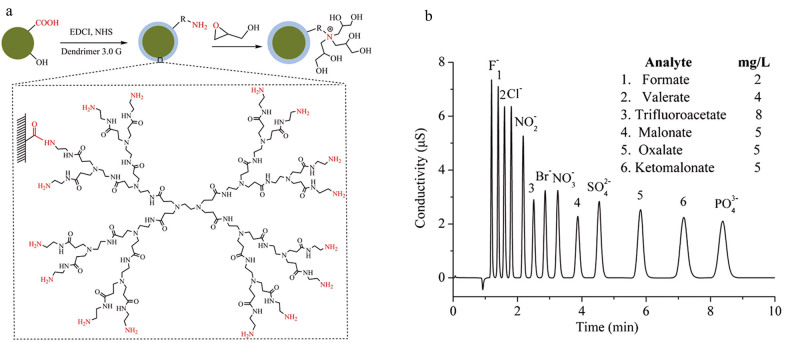
（a）PAMAM接枝型AEC的制备及（b）其对无机阴离子和有机酸的分离应用^［25］^

值得注意的是，尽管PAMAM树状大分子在离子色谱固定相填料的制备中已取得初步应用进展，但在AEC使用强碱性淋洗液的条件下，其酰胺结构的pH耐受性仍存在较大争议。此外，除季铵化末端官能团外，PAMAM树状大分子内部还含有大量的重复结构单元，这些结构单元对离子色谱固定相分离性能的影响机制仍需深入研究和探讨。

## 4 结论与展望

近年来，基于质子化/季铵化PAMAM树状大分子与阴离子之间的静电相互作用机制，PAMAM树状大分子材料在离子型分离材料领域取得了显著进展。这些研究成果不仅拓展了PAMAM材料的应用范围，同时也为具有类似结构和性能的其他树状大分子材料的开发与应用提供了新的研究思路。目前，在功能化吸附剂和色谱分离材料领域，PAMAM树状大分子的应用仍存在诸多发展契机。具体体现在以下3个方面：（1）通过多元化的修饰策略，将磷酸根、羧酸根等阴离子基团引入PAMAM末端，有望开发出高效阳离子交换材料；（2）利用树状大分子优异的生物相容性，将其与石墨烯等生物质材料复合，可为生命科学领域活性带电物质的分离分析提供更为优异的解决方案；（3）PAMAM树状大分子独特的多元官能团结构，在混合模式色谱固定相的可控制备中展现出巨大的潜力。然而，PAMAM树状大分子材料的pH耐受性问题仍是一个亟待解决的问题，这在一定程度上限制了其作为分离材料的应用范围和使用寿命。未来需要通过分离条件的进一步优化或新型制备方法的开发来实现这一问题的有效解决。
